# Inhibition of Type IV Secretion Activity and Growth of *Helicobacter pylori* by Cisplatin and Other Platinum Complexes

**DOI:** 10.3389/fcimb.2020.602958

**Published:** 2020-12-18

**Authors:** Clara Lettl, Franziska Schindele, Giambattista Testolin, Alexander Bär, Tobias Rehm, Mark Brönstrup, Rainer Schobert, Ursula Bilitewski, Rainer Haas, Wolfgang Fischer

**Affiliations:** ^1^ Max von Pettenkofer Institute of Hygiene and Medical Microbiology, Faculty of Medicine, LMU Munich, Munich, Germany; ^2^ German Center for Infection Research (DZIF), Munich Site, Munich, Germany; ^3^ Department of Chemical Biology, Helmholtz Centre for Infection Research, Braunschweig, Germany; ^4^ German Center for Infection Research (DZIF), Hannover-Braunschweig Site, Braunschweig, Germany; ^5^ Organic Chemistry Laboratory, University Bayreuth, Bayreuth, Germany

**Keywords:** *Helicobacter pylori*, type IV secretion system, Cag, cisplatin, protein secretion

## Abstract

Type IV secretion systems are protein secretion machineries that are frequently used by pathogenic bacteria to inject their virulence factors into target cells of their respective hosts. In the case of the human gastric pathogen *Helicobacter pylori*, the cytotoxin-associated gene (Cag) type IV secretion system is considered a major cause for severe disease, such as gastric cancer, and thus constitutes an attractive target for specific treatment options against *H. pylori* infections. Here, we have used a Cag type IV secretion reporter assay for screening a repurposing compound library for inhibitors targeting this system. We found that the antitumor agent cisplatin, a platinum coordination complex that kills target cells by formation of DNA crosslinks, is a potent inhibitor of the Cag type IV secretion system. Strikingly, we found that this inhibitory activity of cisplatin depends on a ligand exchange reaction which incorporates a solvent molecule (dimethylsulfoxide) into the complex, a modification which is known to be deleterious for DNA crosslinking, and for its anticancer activity. We extended our analysis to several analogous platinum complexes containing N-heterocyclic carbene, as well as DMSO or other ligands, and found varying inhibitory activities toward the Cag system which were not congruent with their DNA-binding properties, suggesting that protein interactions may cause the inhibitory effect. Inhibition experiments under varying conditions revealed effects on adherence and bacterial viability as well, and showed that the type IV secretion-inhibitory capacity of platinum complexes can be inactivated by sulfur-containing reagents and in complex bacterial growth media. Taken together, our results demonstrate DNA binding-independent inhibitory effects of cisplatin and other platinum complexes against different *H. pylori* processes including type IV secretion.

## Introduction

The human gastric pathogen *Helicobacter pylori* is responsible for chronic gastritis, peptic ulcer disease, as well as gastric adenocarcinoma and MALT lymphoma, and thus represents one of the leading causes for infection-associated morbidity and mortality worldwide. It has been estimated that more than 4 billion individuals are infected with *H. pylori*, albeit with major regional variations (Hooi et al., 2017), and that more than 800,000 new cases of gastric cancer per year can be attributed to *H. pylori* infection (de Martel et al., 2020). Although consensus treatment strategies are available (Fallone et al., 2016), resistance rates against the therapeutically used antibiotics are increasing to an alarming extent (Savoldi et al., 2018). Because of this, *H. pylori* has been included on the WHO priority list for research and development of new antibiotics (Tacconelli et al., 2018). One possible approach toward novel treatment options is to identify potential inhibitors of important virulence properties, which might be utilized to complement established treatment regimes.

One of the major factors involved in pathogenicity of *H. pylori* is the type IV protein secretion system encoded on the cytotoxin-associated gene (*cag*) pathogenicity island (Backert et al., 2017). The Cag type IV secretion system builds up a remarkable multiprotein complex composed of roughly 20 different proteins, which spans the bacterial inner and outer membranes and is able to translocate the bacterial CagA protein into the cytoplasm of gastric cells (Fischer, 2011; Chung et al., 2019; Hu et al., 2019). Furthermore, the Cag type IV secretion system is involved in inducing a strong proinflammatory response in gastric epithelial cells *via* NF-κB signaling (Zhang et al., 2020). The presence of the *cag* pathogenicity island increases the risk of developing severe disease, particularly of gastric adenocarcinoma (Wroblewski et al., 2010).

Although many studies have addressed the composition of the secretion apparatus and the functions of individual components, and despite the availability of high-resolution structures of the Cag secretion apparatus (Frick-Cheng et al., 2016; Chung et al., 2019; Hu et al., 2019), details of the type IV secretion process are still only poorly understood. For example, the role of pilus-like structures associated with the type IV secretion system, and even their composition, are not clear (Backert et al., 2015; Chang et al., 2018). On the other hand, it is well-established that the Cag system, similar to other type IV secretion systems, contains three different putative ATPases that are all essential for the CagA secretion process. Several studies have reported small-molecule inhibitors with the potential of interfering with the Cag type IV secretion system. One study reported the identification of compounds that were able to reduce formation of secretion system-associated pili, and also type IV secretion itself (Shaffer et al., 2016). Other studies have described small-molecule inhibitors that are able to inhibit one of the ATPases, Cagα (Hilleringmann et al., 2006; Sayer et al., 2014; Arya et al., 2019).

In this study, we have used a recently described CagA translocation reporter assay (Schindele et al., 2016) to screen a small-compound repurposing library for molecules that are able to reduce this type IV secretion activity. Apart from a number of molecules with known or suspected antibacterial activities, we identified two anti-cancer drugs, cisplatin and carboplatin, for which antibacterial effects have been described previously. However, we show in detailed follow-up studies, also including other platinum complexes, that their activity against *H. pylori* does not depend on the typical DNA-binding properties required for anticancer activity of the platinum complexes, and thus differs widely from their activity toward other bacteria.

## Materials and Methods

### Bacterial Strains, Cell Lines, and Culture Conditions

*H. pylori* strains P12 and P12 [TEM-1–CagA] (Schindele et al., 2016) were grown on GC agar plates (Oxoid) supplemented with vitamin mix (1%) and horse serum (Life Technologies; 8%) (serum plates), and cultured for 16 to 60 h in a microaerobic atmosphere (85% N_2_, 10% CO_2_, 5% O_2_) at 37°C. AGS cells were cultivated in RPMI (Gibco) supplemented with 10% FCS (heat-inactivated; Life Technologies). Murine L929 fibroblasts were cultivated in DMEM (Gibco) supplemented with 10% FCS, 2 mM l-glutamine (Gibco) and 1 mM sodium pyruvate (Gibco) at 37°C in a 5% CO_2_ incubator.

### Reagents

The LOPAC^1280^ library was obtained from Sigma (ordering no. LO1280). Cisplatin (no. P4394) and *cis*-dichlorido-bis(DMSO)platinum(II) (no. 767654) were purchased from Sigma. Transplatin was purchased from Alfa Aesar (no. 10472). Stock solutions of all platinum complexes were prepared in DMSO at 200-fold their final concentrations in the assays, except where indicated otherwise. The final DMSO concentration in the assays was thus always 0.5%.

### Antibodies, SDS-PAGE, Immunoblotting, and ELISA

A polyclonal antiserum against the CagA EPIYA region (AK299) has been described previously (Schindele et al., 2016). Sodium dodecyl sulfate–polyacrylamide gel electrophoresis (SDS–PAGE) and Western blotting was performed as described (Fischer et al., 2001). For the development of immunoblots, polyvinylidene difluoride (PVDF) filters were blocked with 5% non-fat milk powder in TBS (50 mM Tris–HCl, pH 7.5, 150 mM NaCl), 0.1% (v/v) Tween 20 (TBS-T), and incubated with the respective antisera at appropriate dilutions in TBS-T with 1% non-fat milk powder. Alkaline phosphatase-conjugated protein A was used to visualize bound antibody. Standard infections of AGS cells with *H. pylori* strains and subsequent preparations for phosphotyrosine immunoblotting were performed as described previously (Odenbreit et al., 2000). Briefly, cells seeded in 6-well plates (Falcon) were infected with bacteria at a multiplicity of infection of 100 for 4 h at 37°C, washed three times and suspended in PBS containing 1 mM Na_3_VO_4_, 1 mM PMSF, 10 μg ml^−1^ leupeptin, and 10 μg ml^−1^ pepstatin. Cells with adherent bacteria were collected by centrifugation and resuspended in SDS-PAGE sample solution. Tyrosine-phosphorylated proteins were analyzed by immunoblotting with the phosphotyrosine antibody PY99 (Santa Cruz Biotechnologies). Production of IL-8 by AGS cells after infection with *H. pylori* strains for 4 h was determined from co-incubation supernatants by a sandwich ELISA as described elsewhere (Fischer et al., 2001). As a control, IL-8 production was induced with 20 ng/ml recombinant human TNF-α (Peprotech Inc.).

### TEM-1–CagA Translocation Assay

The CagA translocation reporter assay with *H. pylori* strains producing TEM-1–CagA fusions was performed as described elsewhere (Schindele et al., 2016). Briefly, AGS cells were co-incubated with *H. pylori* P12 [TEM-1-CagA] for 2.5 h in 384-well, or in 96-well microtiter plates (black, clear bottom, tissue culture treated, 4titude) in PBS/10% FCS. After infection, cells were loaded with the fluorescent substrate CCF4-AM in a loading solution (LiveBLAzer-FRET B/G loading kit; Invitrogen) supplemented with 1 mM probenecid (Sigma) according to the manufacturer´s instructions. For fluorescence quantification by plate reading, infected cells were incubated with this loading solution at room temperature in the dark for 2 h, and then directly measured with a Clariostar reader (BMG Labtech) using an excitation wavelength of 405 nm, and emission wavelengths of 460 nm, or 530 nm. CagA translocation was calculated as the ratio of background-corrected (wells containing no cells or bacteria, but CCF4-AM loading solution) emission values at 460 to 530 nm, which were normalized to P12 [TEM-1–CagA] as a positive control, and P12 [TEM-1–CagA], Δ*cagT* as a negative control. For inhibition experiments, bacteria were pre-incubated with the respective concentrations of compounds obtained from the corresponding stock solutions for 30 min at 37°C in PBS/10% FCS, followed by infection for 2.5 h in the presence of the compound. Additionally, CagA translocation reporter assays were performed in brain heart infusion (BHI, BD), *Brucella* broth (BB, BD, supplemented with 10% FCS) or PBS/10% FCS mixed with the indicated amount of BB. To test the impact of amino acids on the inhibitory effect of cisplatin, stock solutions of l-cysteine (Serva), l-methionine (Merck) and l-alanine (Roth) were prepared in ddH_2_O and mixed with cisplatin (6.3 mM in DMSO). Cisplatin-amino acid mixtures were stored at −20°C until further use.

### Growth Assays

*H. pylori* grown on serum plates were suspended to an optical density (OD_550 nm_) of 0.075 in BB/10% FCS and sub-cultured in 96-well microtiter plates (clear, flat-bottom, Costar, Corning Inc.). Compounds were added to the respective concentrations from corresponding stock solutions, and wells were sealed with a gas-permeable membrane (Breathe-Easy^®^ sealing membrane, Diversified Biotech). Plates were incubated at 37°C, 10% CO_2_, 200 rpm in a plate reader (Clariostar, BMG Labtech) with an atmospheric control unit (BMG Labtech). OD_550_ was automatically measured every 5 min until the stationary phase was reached. Growth curves were analyzed and processed using the MARS Data Analysis software 3.10 R5 (BMG Labtech). The effect of cisplatin on *H. pylori* viability in the presence of AGS cells was further assessed by incubating the bacteria (OD_550 nm_ = 0.1) together with AGS cells in PBS/10% FCS or BB/10% FCS supplemented with 100 µM cisplatin in DMSO, or with DMSO only. After 2 h at 37°C, 10% CO_2_, 5 µl of bacterial suspension were spotted on serum plates, and growth was checked after 24 h.

### Mass Spectrometry and NMR Analysis

LCMS measurements were performed using an HPLC (Agilent technologies 1200 series) equipped with a Gemini-NX 3u C18 110A 50 × 2.0 mm column coupled to an ion trap mass spectrometer (Bruker amaZon SL). High resolution mass spectra were recorded by direct infusion into a Q-TOF mass spectrometer (Bruker maXis HD) using electrospray ionization (ESI) in the positive mode. ^1^H and ^195^Pt NMR spectra were recorded using a Bruker Advance-III HD 700 MHz spectrometer. Chemical shifts are reported as values in ppm, for the ^1^H-NMR relative to residual solvent signal as internal standard and for the ^195^Pt-NMR relative to the reference compound Na_2_PtCl_6_.

### Cytotoxicity Measurements

The effect of compounds on eukaryotic cell proliferation and viability was assessed using the WST-1 cell proliferation assay (Roche Applied Science). Briefly, murine L929 fibroblasts were seeded into 96-well plates (3.0 × 10^5^ cells/well, clear, flat-bottom, Costar, Corning Inc.) using Phenol red-free culture medium. After 24 h incubation at 37°C, 5% CO_2_, compounds were added in two-fold dilutions and incubation was continued for three days. WST-1 reagent was added according to the manufacturer’s protocol and plates were kept at 37°C, 5% CO_2_ for 1 h. The absorbance at 450 nm and 690 nm (reference wavelength) was recorded in a plate reader (Clariostar, BMG Labtech). For evaluation, the difference of A_450nm_ and A_690nm_ was calculated, and the control value (medium and WST-1 only) was subtracted. Percental viability was normalized to the untreated control.

### Adherence Assays

Cisplatin influence on adherence of *H. pylori* to AGS cells was essentially measured as described (Königer et al., 2016). Briefly, cells were infected with a GFP-producing variant of strain P12 (P12 [pHel12::*gfp*]; (Königer et al., 2016)), using an MOI of 60, and cisplatin was added at the time of infection, or 30 min later, from DMSO stock solutions of the corresponding concentrations. AGS cell infection was allowed to proceed until 1 h after infection at 37°C and 5% CO_2_. After three washing steps with PBS, cells with adherent bacteria were collected by EDTA treatment, and analyzed in a flow cytometer (FACS CantoII, BD Biosciences). For analysis, the median fluorescence intensity of non-infected cells was subtracted from that of infected samples.

### Statistical Analysis

Quantitative data sets shown are generally average values resulting from at least three independent experiments, with standard deviations. IC_50_ values were calculated from at least three independent experiments by nonlinear regression using the Graphpad Prism5 software.

## Results

### Identification of Cisplatin and Carboplatin as Inhibitors of the Cag Type IV Secretion System

**Figure 1:**
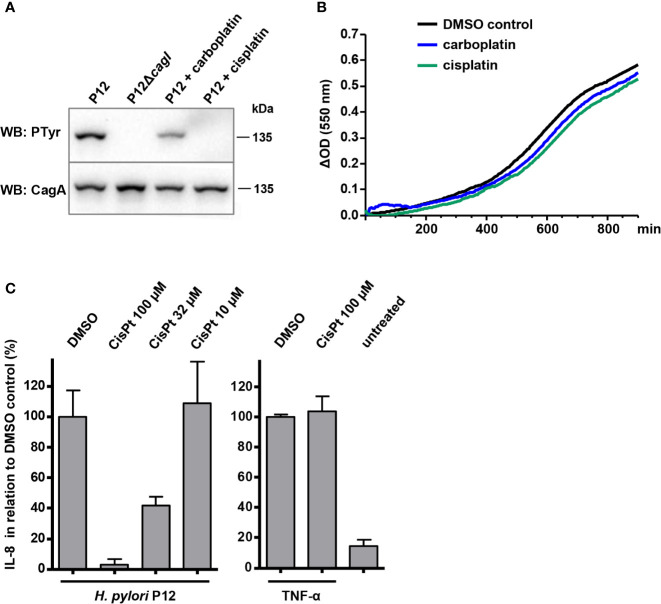
Effects of cisplatin and carboplatin on type IV secretion and on *H. pylori* growth. **(A)** Bacteria were pre-incubated for 30 min at 37°C, 10% CO_2_ in PBS/10% FCS in the presence or absence of the indicated compounds at 50 µM, and subsequently used for co-incubation with AGS cells for 4 h in the same medium. Translocation of CagA was determined by its subsequent tyrosine phosphorylation using phosphotyrosine (PTyr) or CagA immunoblots. **(B)** *H. pylori* P12 was grown in BB/10% FCS in a microtiter plate in the presence of 0.5% DMSO (control), or of the indicated compounds at 50 µM. **(C)** Supernatants of AGS cells co-incubated with *H. pylori* under the same conditions as in **(A)**, but with different amounts of cisplatin (CisPt), were analyzed for IL-8 concentrations by sandwich ELISA (left panel). The indicated values were normalized to the values obtained with strain P12 treated with 0.5% DMSO alone. As a control, AGS cells were treated with 20 ng/ml TNF-α in the presence or absence of 100 µM cisplatin, and IL-8 values were normalized to those obtained for TNF-α without cisplatin (right panel). All data represent mean values of four independent experiments with standard deviations.

In an attempt to identify small molecules that inhibit the Cag type IV secretion system, we screened the Library of Pharmacologically Active Compounds (LOPAC^1280^; Sigma) with a TEM-1–CagA translocation reporter assay (Schindele et al., 2016) adapted to a microtiter plate format. This assay utilizes engineered *H. pylori* strains producing the TEM-1 β-lactamase fused to the N-terminus of CagA. Type IV secretion of the TEM-1–CagA fusion protein into target cells is monitored by loading the cells with the fluorescent β-lactamase substrate CCF4-AM, and CagA translocation is determined by calculating blue-to-green fluorescence ratios (see Experimental Procedures for details). Out of the 1280 compounds tested at a concentration of 50 µM, 151 resulted in a reduction of CagA translocation activity to less than 50%, as compared to infection with an untreated reporter strain. Since these compounds included several substances with suspected or previously demonstrated antibacterial activities against *H. pylori*, such as doxycycline and minocycline (Glupczynski et al., 1988), niclosamide (Tharmalingam et al., 2018), or clotrimazole, we applied a counterscreen for compounds inhibiting *H. pylori* growth. When all compounds that inhibited growth to more than 50% were removed, only 10 compounds remained (**Table 1**). Subsequently, we used the TEM-1–CagA reporter assay in a 96-well format to reproduce the type IV-inhibitory effects of these compounds at a slightly lower concentration (35 µM). In this assay, three compounds exhibited cytotoxic activities toward AGS cells, as indicated by CCF4-AM loading defects, and the initial inhibitory effects could not be reproduced for four further compounds at this concentration (**Table 1**). Thus, only the unspecific alkylating agent iodoacetamide, and the antitumor agents cisplatin and carboplatin remained as potential type IV secretion inhibitors. The activities of the latter compounds were confirmed in an orthogonal secondary assay, which measures CagA tyrosine phosphorylation following infection of AGS cells with *H. pylori* strain P12. While iodoacetamide did not inhibit CagA tyrosine phosphorylation (data not shown), cisplatin and carboplatin were able to strongly reduce or even block the appearance of tyrosine-phosphorylated CagA at a concentration of 50 µM, suggesting that they interfere with type IV secretion of CagA (**Figure 1A**). In contrast, both cisplatin and carboplatin apparently did not interfere with viability of *H. pylori* at these concentrations, as demonstrated by growth curves obtained in *Brucella* broth containing 10% FCS (BB/10% FCS; **Figure 1B**); however, the activity of these compounds depends on the media used (see below). Furthermore, levels of the chemokine interleukin-8 (IL-8), which is induced in AGS cells *via* the Cag type IV secretion system, were reduced in the presence of cisplatin in a dose-dependent manner (**Figure 1C**). In contrast, TNF-α was still able to induce an IL-8 response in AGS cells in the presence of 100 µM cisplatin (**Figure 1C**, right panel), indicating that the cisplatin effect on IL-8 induction was due to inhibition of the bacteria. Taken together, these observations suggested a specific interference of cisplatin and carboplatin with type IV secretion by *H. pylori*.

**Table 1:** Properties of compounds identified during initial screening of the LOPAC^1280^ library^a^.

Compound	Effect on type IV secretion (TEM-1–CagA assay) or *H. pylori* growth (% of untreated control)		
	TEM-1–CagA, 384 well, 50 µM	Growth, 100 µM	TEM-1–CagA, 96 well, 35 µM
Carboplatin	13.7	84.9	32.8
Cisplatin	3.4	88.8	7.6
Cantharidic acid	40.1	84.7	No CCF4 loading
Cantharidin	32.7	74.6	No CCF4 loading
Dihydroergocristine methanesulfonate	39.7	72.5	80.2
Iodoacetamide	47.3	74.7	49.2
Methysergide maleate	5.7	83.3	89.5
NF449 octasodium salt	42.9	102.7	103.0
Propantheline bromide	38.7	65.6	84.0
Wortmannin	35.1	94.0	Low CCF4 loading

^a^Compounds were initially selected for inhibition of TEM-1–CagA activity by more than 50%, and H. pylori growth inhibition by less than 50% in comparison to untreated (DMSO) control.

### Dependence of Cisplatin Activities on the Solvent

**Figure 2:**
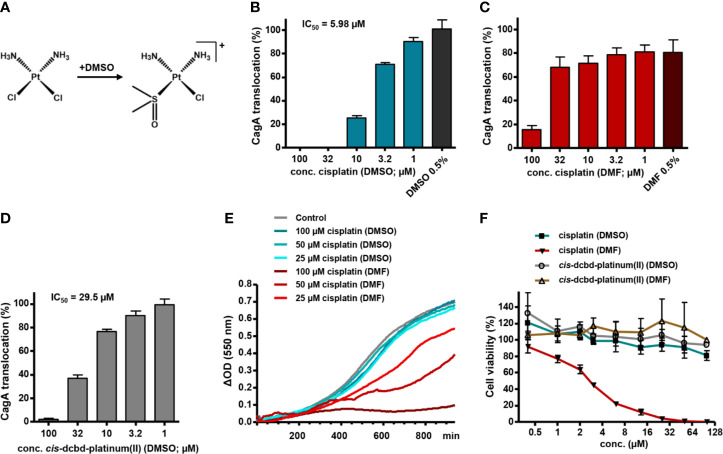
Solvent influence on type IV secretion inhibition and cytotoxicity by platinum complexes. **(A)** Scheme illustrating the potential chlorido to DMSO ligand exchange reaction upon solution of cisplatin in DMSO. **(B)** *H. pylori* P12 producing a TEM-1-CagA fusion was pre-incubated for 30 min with different concentrations of cisplatin diluted from corresponding stock solutions in DMSO (so that the final DMSO concentration was 0.5% in each case), subsequently co-incubated with AGS cells for 150 min, and then analyzed *via* the TEM-1-CagA reporter assay for translocation of CagA. Mean values of inhibition experiments were subjected to nonlinear regression analysis, resulting in the indicated IC_50_ value for CagA translocation inhibition. The indicated bars represent mean values including standard deviations of six independent experiments normalized to P12 [TEM-1-CagA] left untreated, which was set to 100%. **(C, D)** The same experiments as in **(B)** were performed with cisplatin diluted from stock solutions in DMF **(C)**, or with a *cis*-dichlorido-bis(DMSO)platinum(II) complex (*cis*-dcbd-platinum(II)) diluted from stock solutions in DMSO **(D)**. The indicated bars represent mean values including standard deviations of four **(C)**, or seven **(D)** independent experiments, respectively, normalized to P12 [TEM-1-CagA] treated with 0.5% DMSO only, which was set to 100%. Note that addition of 0.5% DMF alone reduces CagA translocation by about 20%. **(E)** Growth curves under standard growth conditions of *H. pylori* P12 in the absence (Control with 0.5% DMSO) or presence of the indicated compound concentrations taken from the respective stock solutions. Representative curves are shown. **(F)** Cytotoxicity of the indicated compounds, diluted to the respective concentrations from stock solutions in either DMSO or DMF, toward L929 cells, as determined by a WST-1 assay. The indicated values represent mean values including standard deviations of three independent experiments normalized to untreated control.

Both cisplatin (*cis*-diamminedichloridoplatinum(II)) and carboplatin (*cis*-diammine-(1,1-cyclobutane-dicarboxylato)-platinum(II)) are planar platinum coordination complexes that are able to form intrastrand crosslinks between purine bases on DNA (Fichtinger-Schepman et al., 1985). For cisplatin, this crosslinking activity is thought to involve prior exchange of at least one chlorido ligand for a water molecule (aquation), which results in a cationic complex with higher affinity for DNA (Davies et al., 2000; Kelland, 2007). Other ligand exchange reactions may also take place, for example the replacement of a chlorido ligand for dimethylsulfoxide (DMSO), which coordinates platinum *via* its nucleophilic sulfur atom (**Figure 2A**) (Annibale et al., 1983). Since the stock solutions of the LOPAC^1280^ library compounds were prepared in DMSO and then diluted in aqueous buffers, such complexes with DMSO ligands may have been present in our screening experiments.

To examine this possibility, we compared the activity of cisplatin dissolved in DMSO with that of cisplatin diluted from stock solutions prepared in dimethylformamide (DMF), which does not seem to react with cisplatin (Hall et al., 2014). With cisplatin dissolved in DMSO, we obtained a dose-dependent inhibition of CagA translocation with a half-maximal inhibitory concentration (IC_50_) of 5.98 µM, as measured by the TEM-1–CagA assay, whereas DMSO had no effect (**Figure 2B**). Using cisplatin from DMF stock solutions, however, we achieved inhibition only at high concentrations (>32 µM), clearly indicating a solvent-dependent activity of cisplatin (**Figure 2C**). We also obtained an inhibition of CagA translocation with *cis*-dichlorido-bis(DMSO)platinum(II) dissolved in DMSO, albeit at a reduced efficiency in comparison to cisplatin (IC_50_ = 29.5 µM; **Figure 2D**), but not when the same complex was dissolved in DMF instead (IC_50_ ≥ 100 µM; data not shown). Interestingly, when we measured *H. pylori* growth in a microplate format, we did not observe significant growth defects upon addition of cisplatin dissolved in DMSO (**Figure 2E**), or *cis*-dichlorido-bis(DMSO)platinum(II) dissolved in DMSO or in DMF (data not shown). However, cisplatin dissolved in DMF resulted in a clear dose-dependent growth inhibition (**Figure 2E**). This suggests that cisplatin dissolved in DMF exerts its expected toxic activity against *H. pylori*, but does not impact strongly on type IV secretion, whereas a DMSO ligand in the platinum complex modulates its activity toward Cag type IV secretion inhibition, while reducing the antibacterial effect.

**Figure 3:**
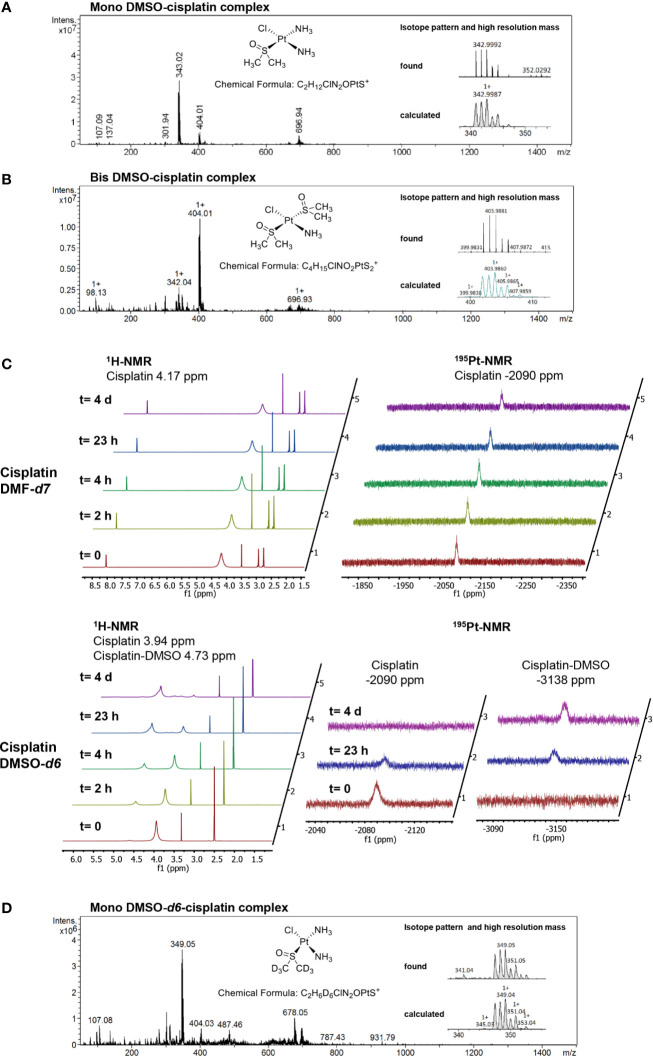
Analysis of platinum complexes formed in different solvents. **(A)** Mass spectrum (MS) and high-resolution mass spectrum (HRMS) showing the correct isotopic pattern of the main component in the sample of cisplatin dissolved in DMSO. **(B)** MS and HRMS showing the isotopic pattern of cisplatin dissolved in DMSO and diluted with water/acetonitrile 70/30 containing 0.1% HCOOH. **(C)** Stacked ^1^H-NMR and ^195^Pt-NMR spectra, recorded at the indicated time points, of cisplatin dissolved either in DMF-*d*_7_ or in DMSO-*d*_6_. In contrast to the DMSO-*d*_6_ solution, cisplatin in DMF-*d*_7_ is stable over several days. In the time between the analyses, the samples were kept at 4°C. **(D)** MS and HRMS spectra of the cisplatin DMSO-*d6* solution analyzed by NMR.

To examine whether cisplatin was subject to ligand exchange reactions in our DMSO stock solutions, we analyzed the complexes by mass spectrometry, and detected a cisplatin derivative with one chlorido ligand replaced by DMSO as the major molecular species in DMSO solution (m/z = 342.999 Da; **Figure 3A**). Upon dilution in water or acetonitrile containing 0.1% formic acid, this complex partly reacted further to give a monoammine-monochloro-bis-DMSO complex (m/z = 403.988 Da; **Figure 3B**). No ligand exchange reactions were observed with cisplatin dissolved in DMF (data not shown). Consistent with these results, ^1^H-NMR and ^195^Pt-NMR measurements of cisplatin dissolved in DMF-*d*_7_ showed stable peaks at 4.17 ppm and −2090 ppm, respectively (**Figure 3C**). In contrast, cisplatin dissolved in DMSO-*d*_6_ was subject to decomposition over time, with formation of a main species within 24 h, as observed by both ^1^H-NMR and ^195^Pt-NMR (**Figure 3C**). Particularly, the signals at time zero at 3.94 ppm and −2090 ppm for the ^1^H and ^195^Pt nuclei, respectively, decreased over time, with a concomitant increase of peaks at 4.73 ppm and −3138 ppm, respectively. Analysis of the NMR sample by mass spectrometry confirmed the exchange of a chlorido ligand for a DMSO-*d*_6_ molecule (**Figure 3D**). Keeping the DMSO-*d*_6_ sample at room temperature showed further conversion of the newly formed complex, which could be avoided by keeping the samples at 4°C between the analyses. At this or lower temperatures, the cisplatin-DMSO complex proved to be stable for at least several days. Taken together, NMR and mass spectrometry data indicate the formation of DMSO adduct derivatives of cisplatin in DMSO-containing solutions.

Interestingly, incorporation of DMSO as a ligand in the platinum complex has been described as a functional inactivation with respect to DNA interaction, since DMSO represents a poor leaving group (Annibale et al., 1983). Therefore, dissolving cisplatin in DMSO is strongly discouraged in order not to lose its cytotoxic activity (Hall et al., 2014). Using a WST-1 cytotoxicity assay in L929 murine fibroblasts, we confirmed that cisplatin dissolved in DMF has a cytotoxic effect, whereas cisplatin dissolved in DMSO, or *cis*-dichlorido-bis(DMSO)platinum(II), dissolved in either DMF or DMSO, showed no cytotoxic effects at the same concentrations (**Figure 2F**). This suggests that the inhibitory effect of cisplatin on type IV secretion is not a consequence of its DNA-binding capability.

### Effects of Other Platinum Complexes on Type IV Secretion Inhibition and *H. pylori* Growth

**Figure 4:**
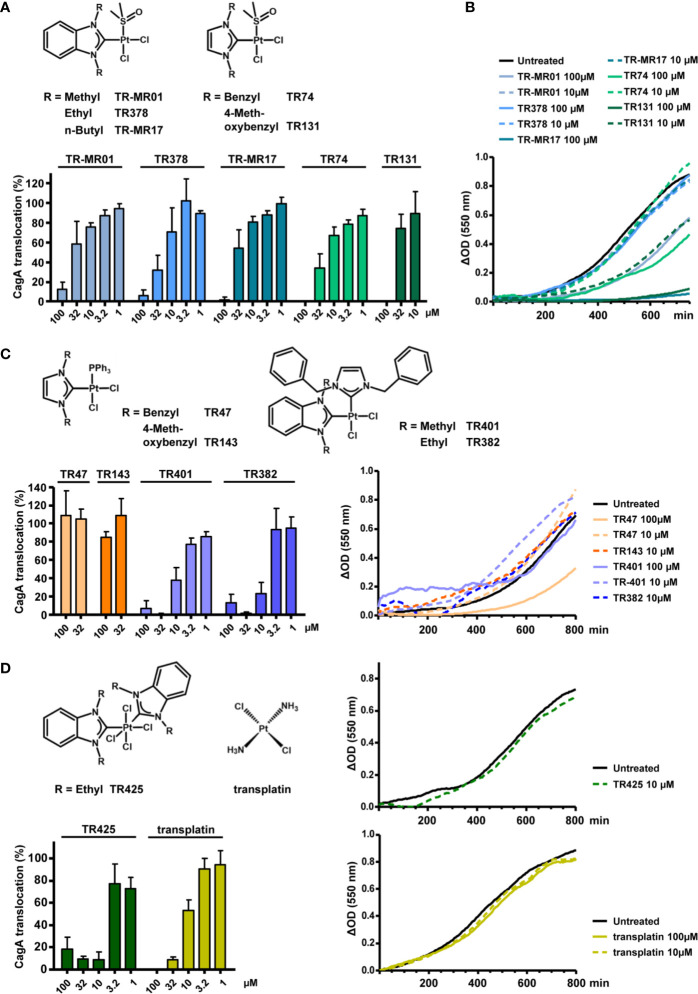
Inhibitory properties of NHC-platinum complexes toward type IV secretion and growth. **(A)** The indicated stable platinum benzimidazol-2-ylidene or imidazol-2-ylidene complexes with DMSO ligands were added to *H. pylori* P12 [TEM-1-CagA] pre-incubation suspensions at the indicated concentrations, and type IV translocation of CagA was determined with the TEM-1-CagA reporter assay. **(B)** The same complexes were added to *H. pylori* P12 suspensions in BB/10% FCS, and optical densities (550 nm) were recorded for 13-14 h in a microtiter plate. Growth curves are shown as optical density differences. **(C)** Analogous complexes with triphenylphosphane ligands instead of DMSO ligands, or complexes containing two different NHC ligands, were added at the indicated concentrations, and examined with the TEM-1-CagA reporter assay as in **(A)** (left panel). Growth curves in the presence or absence of the same complexes at the indicated concentrations (right panel). Some compounds partially precipitated in growth medium when added at 100 µM, and are therefore shown at 10 µM only. **(D)** An octahedral tetrachloridoplatinum(IV) complex with two NHC ligands, or transplatin were analyzed with the TEM-1-CagA assay at the indicated concentrations (left panel) Growth curves in the presence of the same complexes (right panels). Due to partial precipitation at 100 µM, TR425 is only shown at a concentration of 10 µM. All indicated bars represent mean values including standard deviations of at least 3 independent experiments normalized to P12 [TEM-1-CagA] treated with 0.5% DMSO, which was set to 100%. Representative growth curves are shown.

It has been shown before that cisplatin has cytotoxic effects on *E. coli* and other bacteria, particularly if these have defects in their DNA damage repair pathways (Bhattacharya and Beck, 2002), suggesting that the DNA-binding properties of cisplatin are responsible for this finding. To further evaluate whether the observed type IV secretion-inhibitory effects may depend on DNA interaction, we extended our experiments to other platinum complexes with various ligands, structures, and oxidation states of platinum. First, we made use of dichloridoplatinum(II) complexes containing DMSO and N-heterocyclic carbene (NHC) ligands, which exhibit variable DNA-binding properties depending on steric shielding of their leaving groups (Muenzner et al., 2015; Rehm et al., 2018). Different dichloridoplatinum(II) complexes with imidazol-2-ylidene or benzimidazol-2-ylidene ligands in combination with DMSO were found to inhibit CagA translocation, as analyzed with the TEM-1–CagA assay (**Figure 4A**). Some of these compounds had additional effects on *H. pylori* growth, but only at higher concentrations (**Figure 4B**). To examine the role of DMSO in these complexes, we next tested corresponding NHC complexes in which the DMSO ligand is replaced by a triphenylphosphane ligand, a variation which results in slower DNA binding due to increased shielding of the chloride leaving group, and altered responses of cancer cells (Muenzner et al., 2015). Interestingly, those NHC-triphenylphosphane complexes which did not interfere with *H. pylori* growth, also did not substantially inhibit CagA translocation (**Figure 4C**), indicating that the DMSO ligand cannot be functionally replaced by a triphenylphosphane ligand. However, when we tested complexes in which the DMSO ligand was replaced by a second NHC ligand in *cis* configuration (Rehm et al., 2016), we found some with clear antibacterial effects toward *H. pylori* (data not shown), but also others which had similar properties as cisplatin or the DMSO-containing complexes (**Figure 4C**). Finally, we analyzed the effect of an analogous platinum(IV) complex with two NHC ligands, which exhibits reduced DNA-binding properties and only moderate cytotoxicity (Rehm et al., 2019). Here, we also observed a substantial inhibitory potential with respect to CagA type IV secretion for compound TR425, while *H. pylori* growth was not strongly affected (**Figure 4D**). This result confirms that the strength of interaction with DNA does not correlate with type IV secretion inhibition. In the same line, we found that transplatin (*trans*-dichloridodiammineplatinum(II)), which does not show DNA binding-dependent cytotoxicity, but is subject to ligand exchange when dissolved in DMSO (Hall et al., 2014), still inhibits CagA translocation, albeit with a reduced efficiency in comparison to cisplatin (IC_50_ = 10.9 µM; **Figure 4D**).

### Antibacterial Effects of Cisplatin on *H. pylori* and Influence on Adherence

**Figure 5:**
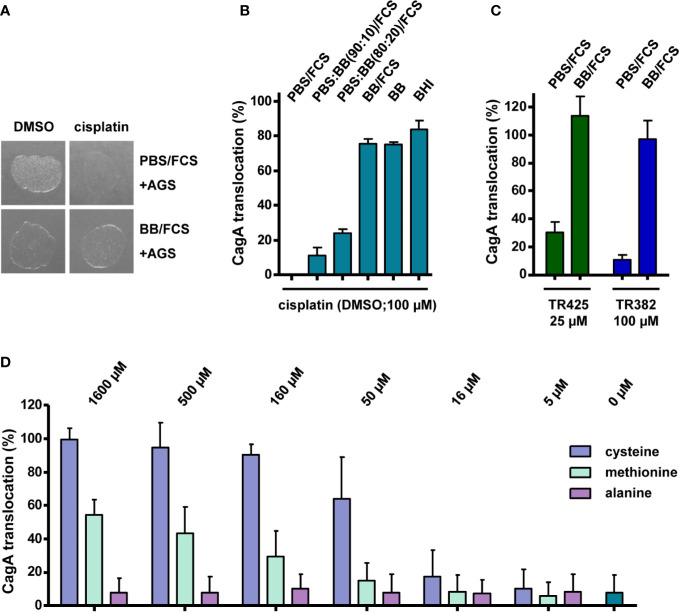
Activities of platinum complexes in different media and during infection of AGS cells. **(A)** *H. pylori* P12 was co-incubated with AGS cells for 2 h with 100 µM cisplatin or 0.5% DMSO in the indicated media, and subsequently spotted on serum agar plates. Growth was monitored after 24 h. **(B)** *H. pylori* P12 [TEM-1-CagA] was pre-incubated and co-incubated with AGS cells in the presence of 100 µM cisplatin in PBS/10% FCS, BB/10% FCS, or mixtures of both, as indicated, and CagA translocation was determined by the TEM-1-CagA reporter assay. The same tests were also performed with BB or BHI medium alone. **(C)** NHC-platinum complexes TR425 and TR382 were added at the indicated concentrations to bacterial suspensions in either PBS/10% FCS, or BB/10% FCS, and CagA translocation was determined after AGS cell infection as in **(B)**. **(D)** *H. pylori* P12 [TEM-1-CagA] was incubated in PBS/10% FCS together with 32 µM cisplatin/DMSO, or additionally with the indicated concentrations of cysteine, methionine, or alanine, and CagA translocation was determined as in **(B)**. All bars represent mean values including standard deviations of at least 3 independent experiments normalized to P12 [TEM-1-CagA] treated with 0.5% DMSO, which was set to 100%.

The results shown so far indicated that some platinum complexes with DMSO or other ligands exert inhibitory effects toward type IV secretion, but do not inhibit bacterial growth, whereas others also gave rise to growth defects. Since measuring growth curves requires the use of complex media, whereas the type IV secretion reporter assay is routinely carried out in phosphate buffer supplemented with fetal calf serum, in which the bacteria do not grow readily, we next tested whether these different media might influence the observed platinum complex effects, in addition to the solvent used for generating stock solutions. When we examined *H. pylori* that had been treated with cisplatin/DMSO in PBS/10% FCS during co-incubation with AGS cells for 2 h, for their viability by plating on standard media, we noticed only weak growth (**Figure 5A**). This antibacterial effect of cisplatin/DMSO was not detected after treatment and co-incubation in BB/10% FCS (**Figure 5A**). Therefore, we carried out the TEM-CagA translocation assay in PBS/10% FCS mixed with different amounts of BB/10% FCS, or in BB/10% FCS alone, which resulted in decreasing inhibition of type IV secretion with increasing amount of BB/10% FCS (**Figure 5B**). A similar loss of inhibitory activity was observed with BB in the absence of FCS, or with BHI growth medium (**Figure 5B**), suggesting that complex media are generally able to inactivate the inhibitory effect of cisplatin. Interestingly, inhibitory platinum(II) or platinum(IV) complexes without DMSO ligands were also subject to inactivation in complex media (**Figure 5C**). Since cisplatin toxicity can be dampened in the presence of thiol- or thioether-containing ligands (Jamieson and Lippard, 1999), we next asked whether thiol-containing reagents might affect the inhibitory activity of cisplatin. Addition of varying concentrations of cysteine to cisplatin solutions before treatment of *H. pylori* resulted in a dose-dependent reduction of the inhibitory potential of cisplatin (**Figure 5D**). A weaker, but similar effect was observed upon addition of different concentrations of methionine, but not with alanine (**Figure 5D**). Taken together, these data strongly suggest that thiol groups may contribute to platinum complex inactivation in complex media.

**Figure 6:**
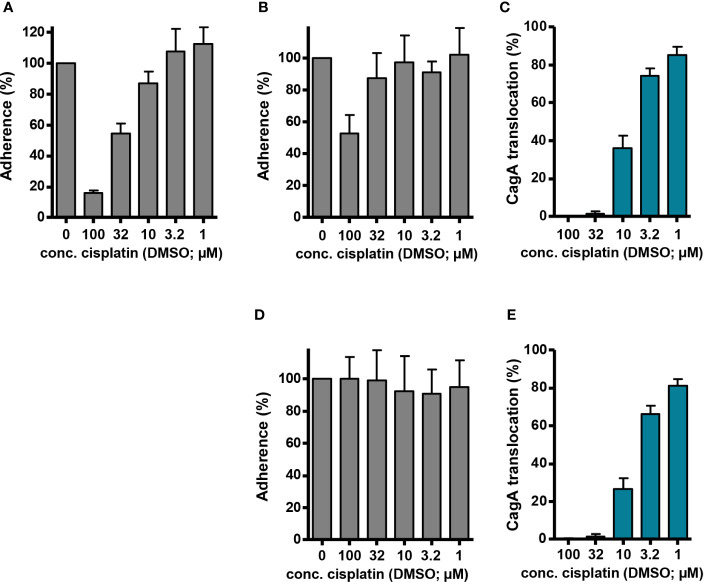
Adherence and CagA translocation in the presence of cisplatin. **(A)** AGS cells were infected with *H. pylori* P12 [pHel12::*gfp*], which had been pre-incubated for 30 min with the indicated concentrations of cisplatin/DMSO (reaching final DMSO concentrations of 0.5% in each case). Fluorescence intensity was determined by flow cytometry after 60 min of infection, and values were normalized to AGS cells that had been infected with P12 [pHel12::*gfp*] treated with 0.5% DMSO only. **(B)** AGS cells were infected at t = 0 with *H. pylori* P12 [pHel12::*gfp*], and cisplatin/DMSO was added at the same time to reach the indicated concentrations at a final DMSO concentration of 0.5%. Adherence was determined, and normalization performed, as in **(A)**. **(C)** AGS cells were infected at t = 0 with P12 [TEM-1–CagA], and cisplatin/DMSO was added at the same time to reach the indicated concentrations at a final DMSO concentration of 0.5%. CagA translocation was determined by the TEM-1–CagA reporter assay after 2.5 h of infection. The indicated values are normalized to P12 [TEM-1–CagA] treated with 0.5% DMSO. **(D, E)** AGS cells were infected at t = 0 with either *H. pylori* P12 [pHel12::*gfp*] **(D)**, or P12 [TEM-1–CagA] **(E)**, and cisplatin/DMSO was added at t = 30 min to reach the indicated concentrations at a final DMSO concentration of 0.5%. Bacterial adherence **(D)**, and CagA translocation **(E)** was determined with normalization as before.

Thus, our original observation that cisplatin/DMSO acts as an inhibitor of Cag type IV secretion might be an indirect consequence of its antibacterial activity. To examine possible indirect effects on type IV secretion further, we determined the influence of cisplatin on *H. pylori* adherence to AGS cells, which is a prerequisite for CagA translocation. To do so, we infected AGS cells with P12 [pHel12::*gfp*], and measured adherence after 1 h of co incubation by flow cytometry. After pre-incubation of the bacteria with cisplatin/DMSO for 30 min, we saw in fact a dose-dependent inhibition of adherence (**Figure 6A**), albeit not as strong as the reduction in CagA translocation (compare with **Figure 2B**). However, when we added cisplatin/DMSO without pre-incubation, i.e. at the same time when the AGS cells were infected with *H. pylori*, adherence was only inhibited at the highest concentration (**Figure 6B**). Nevertheless, cisplatin/DMSO still caused a clear inhibition of CagA translocation under these conditions, as measured with the TEM-1–CagA assay (**Figure 6C**). This difference was even more pronounced when cisplatin was added 30 min after starting the co-incubation of *H. pylori* with AGS cells. In this case, adherence could not be reduced by cisplatin any more, suggesting that established adherence is not disturbed by cisplatin (**Figure 6D**), whereas CagA translocation was still inhibited to a similar extent as before (**Figure 6E**). These results show that type IV secretion inhibition is not just caused by preventing bacteria from establishing secretion-competent cell contacts, and indicate that cisplatin is able to rapidly interfere even with a running secretion process.

## Discussion

Since the original discovery of its antibacterial and its anticancer activities (Rosenberg et al., 1965; Rosenberg et al., 1969), cisplatin, and also other platinum complexes, such as carboplatin or oxaliplatin, have been thoroughly studied with respect to their DNA-damaging and growth-arresting effects on cancer cells (Jamieson and Lippard, 1999). The antibacterial activity has been studied further in *E. coli* with a focus on the influence of DNA damage repair (Beck and Brubaker, 1973; Bhattacharya and Beck, 2002; Calmann and Marinus, 2005; Nowosielska and Marinus, 2005), but a number of studies have also addressed growth-inhibitory effects in other bacteria (Joyce et al., 2010; Chowdhury et al., 2016; Yuan et al., 2018). Similar to the activity against cancer cells, these effects are likely to depend on cisplatin-induced DNA damage, which is supported by the observation that phage induction occurs in *E. coli* upon compound administration (Johnstone et al., 2014; Brabec et al., 2015). Therefore, typical ligand exchange reactions should be required, such as aquation reactions to form cationic diammine-chloro-aqua and diammine-diaqua complexes, which show a higher affinity to DNA (Hardie et al., 2016). Consequently, the effects would be sensitive to prior solubilization of the compounds in DMSO, which can replace chlorido ligands on platinum centers, but represents an inferior leaving group in comparison to chlorido or aqua ligands (Hall et al., 2014).

On the contrary, we show here that the inhibitory effect of cisplatin on Cag type IV secretion by *H. pylori* is not reduced, but rather enhanced, after dissolving cisplatin in DMSO. Our results confirm earlier data (Hall et al., 2014) that a chlorido to DMSO ligand exchange takes place under these conditions, and that a second DMSO molecule might subsequently replace one of the ammine ligands. In comparison to cisplatin dissolved in DMF or directly in aqueous solution (data not shown), this ligand exchange enhances inhibition of type IV secretion and apparently reduces antibacterial activity against *H. pylori*, although the latter effect might be an indirect consequence of a higher sensitivity of the complex for inactivation. Several further observations argue against an involvement of DNA binding in inhibition of Cag type IV secretion by platinum complexes: First, type IV secretion inhibition is very rapid, as determined by the TEM-1–CagA translocation assay. We observed an effect already 30 min after compound exposition, and CagA translocation could be strongly reduced when the compound was administered at the same time with, or even 30 min after, co-incubating the bacteria with gastric epithelial cells. DNA crosslinking may cause defects in bacterial cell division, as suggested by bacterial cell filamentation (Johnstone et al., 2014), but is unlikely to explain such rapid inhibitory effects. Second, the inhibitory effects of different platinum complexes did not correlate with their DNA-binding capability, or cancer cell toxicity. For example, the platinum(IV) complex TR425, which is distinctly less toxic to cancer cells than cisplatin, and binds only weakly to linear DNA (Rehm et al., 2019), had a similar inhibitory potency as cisplatin in our translocation assay. Third, complexes that are considered non-functional in terms of DNA-binding and anticancer activity, such as transplatin, still showed type IV secretion inhibition to a higher extent than cisplatin dissolved in DMF. Collectively, these observations argue for a mode of action of the platinum complexes that is independent of DNA crosslinking.

DNA crosslinking is considered the major mechanism involved in cisplatin anticancer activity, but it is also known that platinum(II) complexes react with proteins in serum or in target cells, notably with cysteine-containing proteins, for instance with copper transporters and copper-binding proteins (Boal and Rosenzweig, 2009). Mutation of such copper transporters in fact results in cellular resistance against cisplatin (Wang and Lippard, 2005). Another mechanism of cisplatin resistance arises from high intracellular levels of glutathione (Jamieson and Lippard, 1999; Kelland, 2007). Whereas replacement of chlorido for amine ligands, such as the guanine N7 atoms on DNA, requires prior aquation of cisplatin, which represents a rate-limiting step (Davies et al., 2000; Hardie et al., 2016), sulfur-containing molecules and residues such as cysteine or methionine can react without prior aquation (Kozelka, 2009; Wexselblatt et al., 2012). Platinum(II) complexes with thiols or thioethers are also more stable than amine complexes (Appleton, 1997). Therefore, DNA binding-independent modes of action of cisplatin are likely caused by protein coordination of the platinum center *via* cysteine or methionine residues, which is also supported by our observation that cysteine and methionine inactivate type IV secretion inhibition by cisplatin. In line with this, it has been described that cisplatin is active against inteins, which rely on essential cysteine residues, in *Mycobacterium* spp. (Zhang et al., 2011; Chan et al., 2016). An inhibitory effect of cisplatin was also shown against anthrax toxin, independently of the bacteria or their DNA (Moayeri et al., 2006); interestingly, in this case, cisplatin activity could be demonstrated using stock solutions prepared in DMSO.

Inhibition of cysteine-containing proteins might also be causative for the type IV secretion inhibition observed here. That cysteine residues and disulfide bond formation are likely involved in the type IV secretion process, is indicated by the deficiency of an *H. pylori* deletion mutant in the disulfide bond oxidoreductase gene *hp0231* for CagA translocation (Zhong et al., 2016). Furthermore, the outer membrane protein HopQ, which plays an important role in the translocation process, requires disulfide bonds for its interaction with CEACAM receptors (Hamway et al., 2020). However, a *hopQ* mutant of strain P12 is still able to translocate reduced levels of CagA into AGS cells (Zhao et al., 2018), and even this reduced translocation could be inhibited by adding cisplatin (data not shown), arguing against a major role of HopQ in the type IV secretion inhibition observed here. Moreover, although we did observe reduced adherence levels in the presence of higher concentrations of cisplatin, this cannot explain the degree of CagA translocation inhibition, particularly under conditions where cisplatin was added after *H. pylori* had already established binding to AGS cells. Nevertheless, as the bacteria did not grow readily after treatment with platinum complexes in PBS/FCS, we cannot fully exclude that the inhibitory effect on type IV secretion is indirect. However, the possibility of interfering with type IV secretion after bacteria-target cell contacts are established, and the fast inhibition argue for a more direct interference with the type IV secretion process. The striking observation that cisplatin dissolved in DMSO, and also some of the NHC complexes, had high type IV-inhibitory activity, but were more readily inactivated in growth media than cisplatin dissolved in DMF, and thus did not cause pronounced growth-inhibitory effects in these media, points to the possibility of generating modified platinum complexes with more selective inhibitory potential and less cytotoxicity. It is not clear at this point, however, whether DMSO-containing platinum complexes are sufficiently stable *in vivo*, and whether the lower cytotoxicity in comparison to cisplatin formulated for classical therapeutic use translates to the *in vivo* situation. Further studies are therefore clearly required to explore this possibility. In any case, our results show that repurposing of approved drugs for inhibition of *H. pylori* might generally be a promising strategy.

## Data Availability Statement

The original contributions presented in the study are included in the article/supplementary material. Further inquiries can be directed to the corresponding author.

## Author Contributions

RH and WF contributed to conception and design of the study. CL, FS, and GT performed the experiments, and MB, RS, and WF supervised the experiments. CL, FS, GT, MB, and WF analyzed, and visualized the data. AB, TR, RS, and UB provided compound resources. MB, UB, RH, and WF contributed to funding acquisition. WF wrote the original draft of the manuscript. All authors contributed to the article and approved the submitted version.

## Funding

This work was supported by a grant from the German Center for Infection Research (DZIF), TTU Gastrointestinal Infections. The funders had no role in study design, data collection and interpretation, or the decision to submit the work for publication.

## Conflict of Interest

The authors declare that the research was conducted in the absence of any commercial or financial relationships that could be construed as a potential conflict of interest.
